# Global research trends on fibromyalgia and exercise: a ten-year Web of Science-based bibliometric analysis

**DOI:** 10.1007/s00296-025-05807-5

**Published:** 2025-02-13

**Authors:** Hilmi Erdem Sumbul, Ramazan Azim Okyay, Dana Bekaryssova, Burhan Fatih Kocyigit

**Affiliations:** 1 Department of Internal Medicine, University of Health Sciences, Adana Health Practice and Research Center, Adana, Türkiye; 2 Faculty of Medicine, Department of Public Health, Kahramanmaraş Sütçü İmam University, Kahramanmaraş, Türkiye; 3 Department of Biology and Biochemistry, South Kazakhstan Medical Academy, Shymkent, Kazakhstan; 4 Department of Physical Medicine and Rehabilitation, University of Health Sciences, Adana City Research and Training Hospital, Adana, Türkiye

**Keywords:** Bibliometrics, Bibliometric analysis, Fibromyalgia, Exercise, Rehabilitation

## Abstract

Fibromyalgia causes widespread pain, exhaustion, and cognitive deficits, lowering sufferers’ quality of life. Exercise supports the management of fibromyalgia by reducing pain and improving mood. This study examines global fibromyalgia and exercise research trends using bibliometric analysis to identify major contributors, citation patterns, and prospective research areas. Data were obtained from the Web of Science (WoS) database utilizing the keywords “fibromyalgia exercise” for publications from 2014 to 2023. The inclusion criteria prioritized original articles and reviews published in the English language. Bibliometric characteristics were examined, including publication year, country, journal, and citation metrics. Statistics adjusted for population and gross domestic product (GDP) were computed to evaluate research productivity in relation to economic and demographic variables. A total of 497 publications satisfied the inclusion criteria. A significant increase trend in publication counts was noted (*p* = 0.003), with Spain (25.75%), the United States (15.09%), Brazil (13.88%), Türkiye (7.24%), and Sweden (5.23%) identified as the major contributors. Publications were produced by 37 countries, 19 of which were the main active countries. Spain displayed remarkable productivity, ranking first in population- and GDP-adjusted contributions. Based on publication type, 388 (78.06%) were original articles, and the rest were reviews. The median number of original article and review citations were 11 (min = 0; max = 289) and 14 (min = 0; max = 1092). Review citations outnumbered original articles (*p* = 0.013). The median number of citations for SCIE and/or SSCI and ESCI articles were 12 (min = 0; max = 1092) and 3 (min = 0; max = 92). SCIE and/or SSCI articles were significantly more cited than ESCI ones (*p* < 0.001). INT J ENV RES PUB HE (*n* = 18), RHEUMATOL INT (*n* = 17), ARCH PHYS MED REHAB (*n* = 15), J CLIN MED (*n* = 14) and DISABIL REHABIL (*n* = 13) were the top five journals in terms of article count. This bibliometric analysis evaluates and summarizes global scholarly output on fibromyalgia and exercise, underscoring the increasing research interest in the two. High-income countries, notably Spain, the United States, and Sweden, significantly contributed to the area, underscoring differences in research capacities.

## Introduction

Fibromyalgia is a chronic condition marked by extensive musculoskeletal pain, exhaustion, sleep disruptions, and cognitive impairments, commonly known as ‘fibro fog’ [1, 2]. The disorder is more common in women and is linked to a heightened sensitivity to pain stimuli, which is probably attributed to changes in the mechanisms that regulate pain centrally [3, 4]. Although the precise cause of fibromyalgia is still unknown, it is generally acknowledged that it is a complex disorder that is influenced by a variety of factors, including genetic, environmental, and psychological components [5]. Fibromyalgia’s impact transcends physical symptoms, considerably diminishing sufferers’ quality of life, psychological well-being, and daily activities. Efficient medical care for this intricate disorder necessitates a multidisciplinary strategy customized to the specific requirements of each patient [6].

Exercise is essential in managing fibromyalgia since regular exercise has been demonstrated to reduce pain, enhance sleep quality, improve mood, and strengthen physical condition [7–9]. Aerobic exercises, strength training, and mind-body approaches, including yoga and tai chi, have all shown beneficial results. Notwithstanding its established benefits, compliance with exercise regimens remains problematic due to apprehension of pain intensification and insufficient customized instruction [10].

Bibliometric analysis has become a critical tool for assessing trends and patterns in scientific research. Bibliometric studies methodically analyze publications, citation indicators, and networks of collaboration to elucidate the growth of a research topic, identify major contributors, and underscore notable Works [11–13]. These inspections are especially useful in pinpointing research gaps and guiding subsequent investigations. Bibliometric studies can elucidate global research endeavors and identify significant contributions in fibromyalgia and exercise. These insights have significance in enhancing understanding and strengthening therapeutic practice in diagnosing and managing fibromyalgia [14].

This bibliometric study analyzes global research trends regarding fibromyalgia and exercise, identifies main contributors, and assesses citation patterns. By evaluating the contributions of countries, journals, and research outputs, it aims to deliver an in-depth overview of advancements in this topic.

## Methodology

### Data source and search strategy

**Fig. 1:**
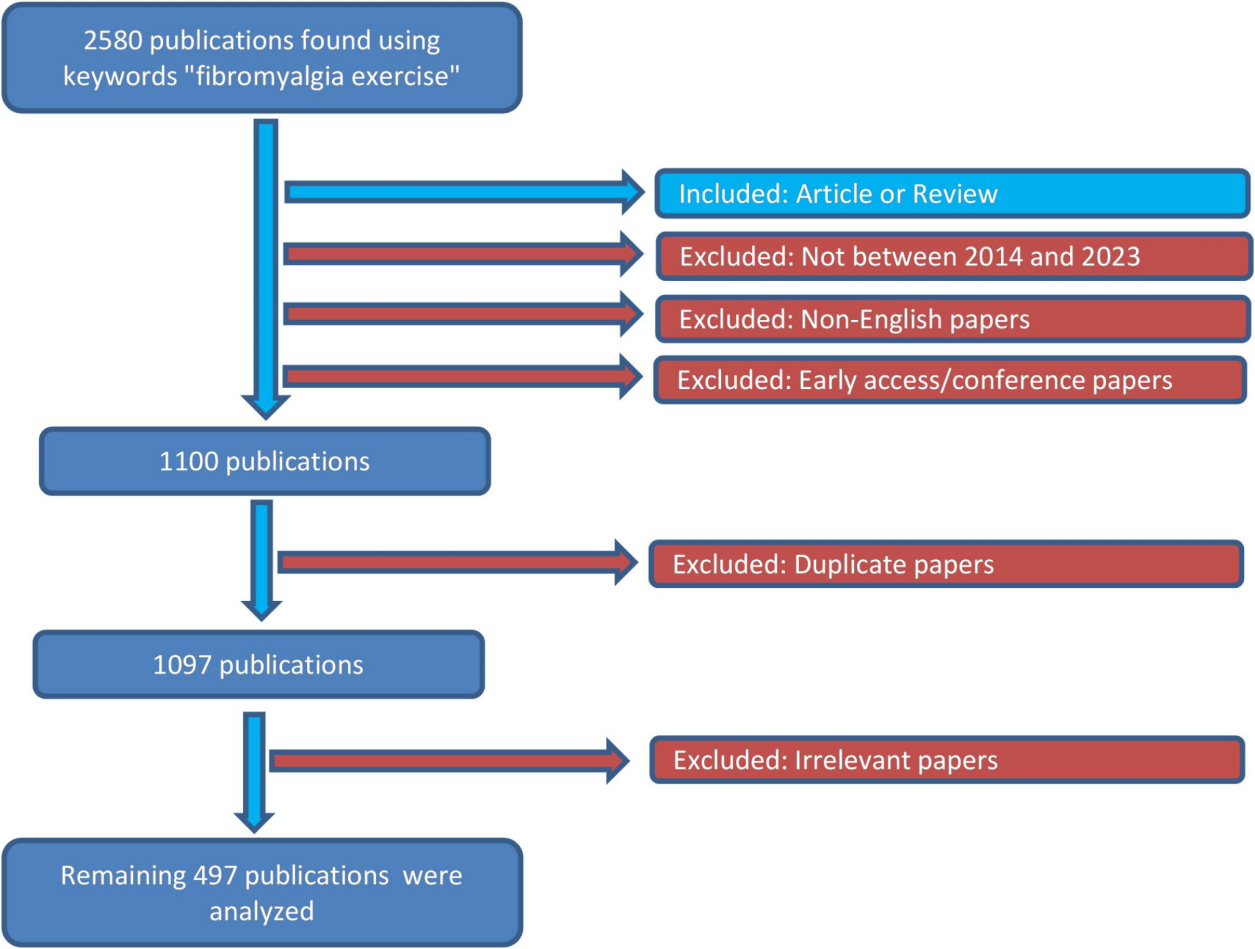
Selection process flowchart

Data were retrieved from the Web of Science (WoS) database. The WoS is a trustworthy database researchers commonly use to acquire citation statistics and other information regarding academic influence. Moreover, this database was chosen since it has been utilized in numerous bibliometric studies comparable to those published in the literature [15–17]. The search was conducted using the keywords “fibromyalgia exercise” without any filters applied. The time frame that was considered was from January 2014 to December 2023. This search yielded a total of 2,580 publications. The inclusion and exclusion criteria were implemented in the selection process flowchart, ensuring a transparent and replicable methodology (Fig. 1). The selection process involved the inclusion of publications categorized as original articles or reviews, published between 2014 and 2023 and written in English. Exclusions were applied to non-English publications, early access or conference papers, duplicate publications, and irrelevant publications based on evaluating titles and abstracts [18]. After applying these criteria, 497 publications were deemed eligible for the bibliometric analysis. Additionally, VOSviewer software was used for bibliometric analysis and visualization.

### Data Analysis

The publications were documented by two researchers (HES and RAO). The total number of articles, publication year, originating country, and citation data were obtained. We adopted this approach to identify the countries contributing the most to the field and their publication trends. Additionally, publications were classified according to journal indexing categories (SSCI, SCIE, and ESCI) and their quartiles (Q1 to Q4). From 2014 to 2023, an analysis was performed on the total number of papers published each year. The procedure employed in similar studies was utilized to determine the country of studies that included authors from various countries, and the corresponding author’s country was regarded as the country of the article [19]. According to the information obtained from ‘https://www.cia.gov/the-world-factbook/field/population/country-comparison/’ and ‘https://www.cia.gov/the-world-factbook/field/real-gdp-purchasing-power-parity/country-comparison/’ the population size and gross domestic product (GDP) values of each country were extracted, and the number of articles was appraised based on these statistics.

Countries contributing 1% or more of the total publications within the specified date range were classified as the main active countries in the literature [20, 21]. A calculation was conducted to determine the total number of papers and citations contributed by each country. The total number of citations was divided by the total number of articles, which calculated the average citation count for each country. The World Bank classified the countries into four categories: high-income, upper-middle-income, low-middle-income, and low-income. These categories were used to classify the countries (https://datatopics.worldbank.org/world-development-indicators/the-world-by-income-and-region.html). Through the use of the formula “number of articles per country/total number of articles,” researchers determined the contribution rate of the main active countries.

The top five journals and five most productive countries were identified based on the number of publications. Researchers identified the top five journals’ leading five countries using article counts.

The leading five institutions, funding organizations, and prolific authors were identified based on publication counts.

The most frequently employed author keywords were derived from VOSviewer software (version 1.6.20). Visualizations were generated based on the analyses of country co-authorship, author co-authorship, and author keyword co-occurrence. In VOSviewer, visualization maps are generated by aggregating countries, authors, journals, or keywords that possess a specific quantity of connections. The line thickness between items indicates the relative strength of their link, with a thicker line denoting a stronger connection. Moreover, distinct groups are created and displayed in analogous colour schemes [22].

### Ethical considerations

This study utilized publicly available bibliometric data from the Web of Science database. Ethical approval was not required as no human or animal subjects were involved.

### Statistical analysis

For statistical analysis, normality tests were conducted to determine data distribution. The Mann-Whitney U test was applied for group comparisons where normality assumptions were not met. A linear regression analysis was used to evaluate the trend in annual publication counts, providing insights into the growth of research over time. The median number of citations and citation distributions for articles and reviews were calculated. Citations were also analyzed based on journal indexing (SCIE/SSCI vs. ESCI) to identify differences in citation impact.

## Results

**Fig. 2:**
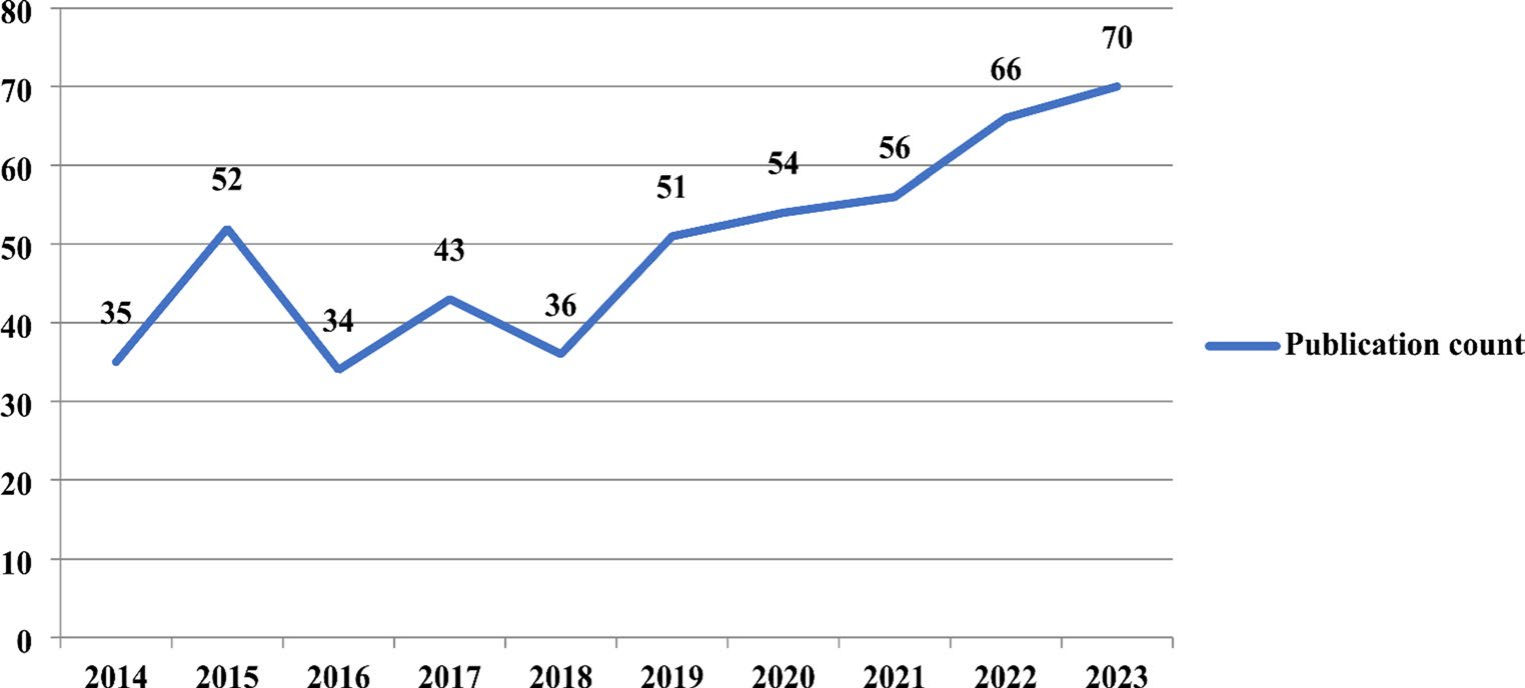
Publication count by year

**Fig. 3:**
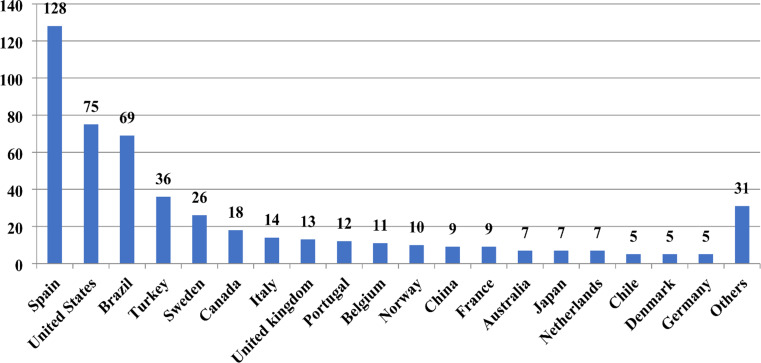
Publication counts of main active countries

Figure 2 shows that the publication count is increasing yearly, peaking in 2023. A linear regression analysis was made to assess the trend. A consistent upward trend in publications was observed, with an average increase of 3.46 publications per year. The analysis showed statistical significance (*p* = 0.003), with the model explaining almost 69% of the variation in publication counts (R^2^ = 0.687). There were a total of 37 countries from which the publications originated. The 37 countries were classified according to World Bank income categories as follows: 4 lower-middle income, 7 upper-middle income, and 26 high income. Among them, 19 were the main active countries, contributing 462 (92.95%) publications (Fig. 3). While 3 main active countries were upper-middle-income, 16 were high-income countries. No main active county fell into the low or lower-middle-income category.

When the publications were evaluated according to document type, 388 (78.06%) were original articles, whereas the remainder were reviews. The median number of citations for articles and reviews were 11 (min = 0; max = 289) and 14 (min = 0; max = 1092), respectively. Reviews were significantly more cited than articles (*p* = 0.013).

Out of the total of 497 journals, 4.02% (*n* = 20 journals) were indexed solely in SSCI, 31.19% (*n* = 155) were indexed both in SSCI and SCIE, 52.72% (*n* = 262) were indexed solely in SCIE. Lastly, 12.07% (*n* = 60) were indexed in ESCI. The journal quartile distribution of journals indexed in SCIE and/or SSCI was as follows: 191 Q1 journals (43.81%), 167 Q2 journals (38.3%), 57 Q3 journals (13.07%), and 21 Q4 journals (4.82%).

The median number of citations were 12 (min = 0; max = 1092) and 3 (min = 0; max = 92) for articles indexed in SCIE and/or SSCI, and ESCI, respectively. Articles indexed in SCIE and/or SSCI were significantly more cited than those indexed in ESCI (*p* < 0.001).

**Fig. 4:**
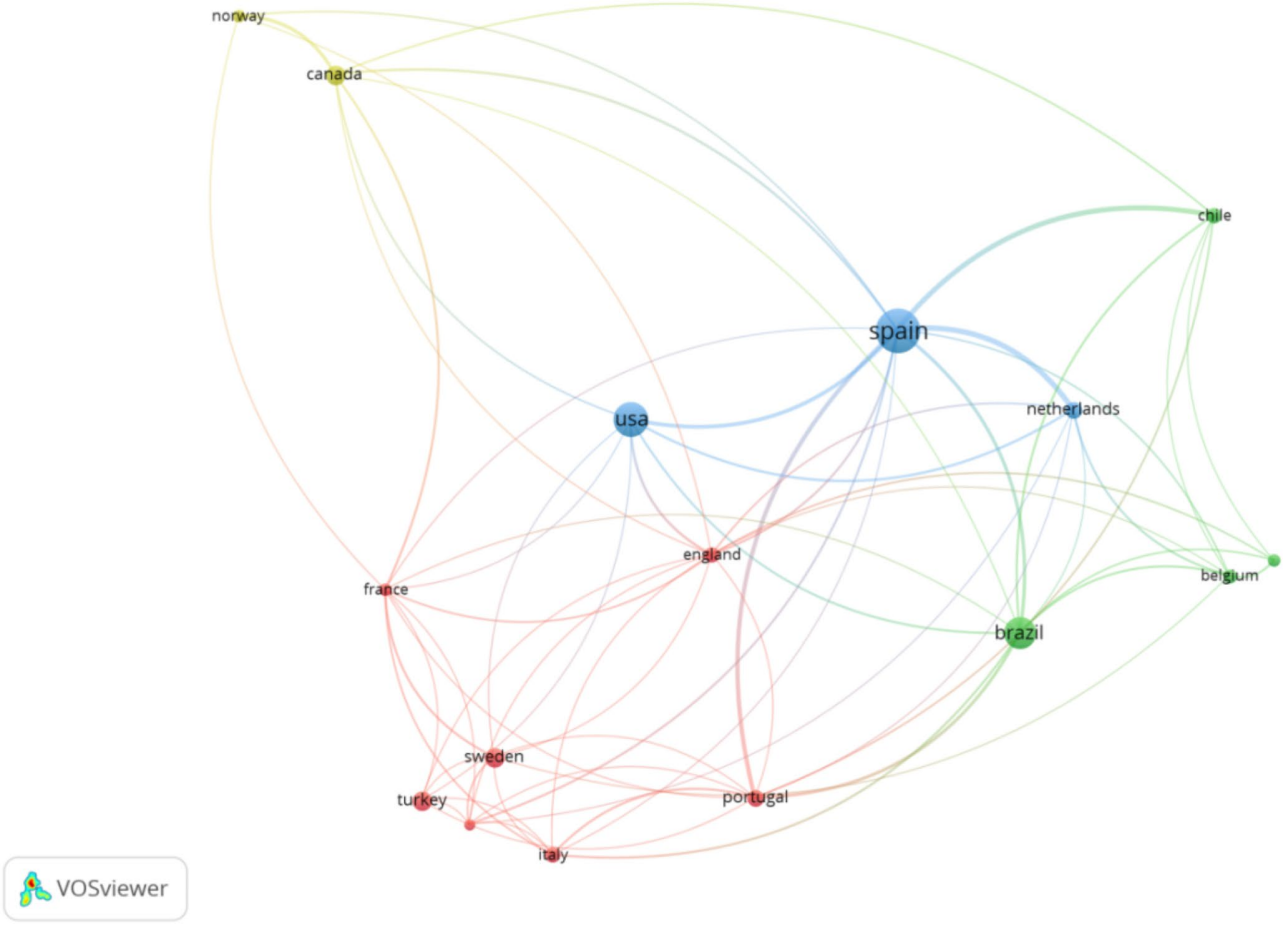
Country co-authorship network visualization map. Four clusters were created. Cluster 1 (Denmark, England, France, Italy, Portugal, Sweden, Turkey) is presented in red; Cluster 2 (Australia, Belgium, Brazil, Chile) is presented in green; Cluster 3 (Netherlands, Spain, The United States of America) is presented in blue; and Cluster 4 (Canada, Norway) is presented in light green

The contribution rates of the top five countries varied annually. Spain ranged from 14.7 to 37.0%, the United States from 4.3 to 35.3%, Brazil from 2.9 to 27.8%, Türkiye from 0.0 to 14.3%, and Sweden from 2.0 to 14.7% (lowest to highest values) (Table 1). A country co-authorship visualization map representing international collaborations was created. Articles with authors from more than 25 different countries were omitted, and the full counting method was selected. A country’s minimum number of articles and citations was set to 10 and 50, respectively. These criteria were met by 16 countries. Weighting was performed according to the number of publications, and the circle size on the map represents the number of articles (Fig. 4).

**Table 1:** Contribution rate of top five countries over the years

	Top five countries				
Years	Spain (%^a^)	United States (%^a^)	Brazil (%^a^)	Türkiye (%^a^)	Sweden (%^a^)
2014	20.0	25.7	5.7	5.7	2.9
2015	30.8	28.8	3.8	7.7	5.8
2016	14.7	35.3	2.9	11.8	14.7
2017	25.6	7.0	14.0	9.3	9.3
2018	16.7	25.0	27.8	0.0	8.3
2019	25.5	9.8	19.6	3.9	2.0
2020	37.0	9.3	16.7	5.6	5.6
2021	26.8	14.3	19.6	5.4	3.6
2022	27.3	9.1	13.6	6.1	3.0
2023	24.3	4.3	12.9	14.3	2.9

^a^Row percentage

Spain contributed 25.75% of publications, followed by the United States (15.09%), Brazil (13.88%), Türkiye (7.24%), and Sweden (5.23). Average citations per publication were highest for the United Kingdom (107.08), followed by the United States (39.48), Norway (34.60), Germany (33.40), and Belgium (30.18). The quantity of articles was adjusted per million population and GDP. The highest values per million population were recorded in Spain (2.71), Sweden (2.46), and Norway (1.81), respectively. According to GDP analysis, Spain (5.71), Sweden (3.84), and Portugal (2.73) occupied the top three positions (Table 2).

**Table 2:** Contribution of main active countries

	*n* (%)	*n* ^a^	*n* ^b^	Total citations	Average citations
Spain	128 (25.75)	2.71	5.71	2067	16.15
United States	75 (15.09)	0.22	0.30	2961	39.48
Brazil	69 (13.88)	0.31	1.72	1210	17.54
Türkiye	36 (7.24)	0.43	1.23	470	13.06
Sweden	26 (5.23)	2.46	3.84	625	24.04
Canada	18 (3.62)	0.46	0.80	539	29.94
Italy	14 (2.82)	0.23	0.45	200	14.29
United Kingdom	13 (2.62)	0.19	0.35	1392	107.08
Portugal	12 (2.41)	1.18	2.73	86	7.17
Belgium	11 (2.21)	0.92	1.46	332	30.18
Norway	10 (2.01)	1.81	2.00	346	34.60
China	9 (1.81)	0.01	0.03	98	10.89
France	9 (1.81)	0.13	0.24	140	15.56
Australia	7 (1.41)	0.26	0.44	199	28.43
Japan	7 (1.41)	0.06	0.12	24	3.43
Netherlands	7 (1.41)	0.39	0.56	154	22.00
Chile	5 (1.01)	0.27	0.86	49	9.80
Denmark	5 (1.01)	0.84	1.17	112	22.40
Germany	5 (1.01)	0.06	0.10	167	33.40

n^a^ number of articles per million population n^b^ number of articles per $ 100 billion gross domestic product

Spain’s highest contributions were to INT J ENV RES PUB HE and J CLIN MED (13 publications each). The United States primarily published in ARTHRIT CARE RES, and CLIN J PAIN (3 publications each). Brazil’s leading contributions were to ADV RHEUMATOL, EUR J PHYS REHAB MED, J BODY MOV THER, REUMATISMO, and TRIALS (3 publications each). Türkiye’s highest publications were in RHEUMATOL INT (5 publications) and TURK J PH MED REHAB (4 publications), and Sweden’s most active journals were ARTHRITIS RES THER and BMC MUSCULOSKEL DIS (4 publications each) (Table 3).

**Table 3:** Top five journals in the five most productive countries

	Top five countries				
Rank	Spain (n)	United States (n)	Brazil (n)	Türkiye (n)	Sweden (n)
1	INT J ENV RES PUB HE (13)	ARTHRIT CARE RES (3)	ADV RHEUMATOL (3)	RHEUMATOL INT (5)	ARTHRITIS RES THER (4)
2	J CLIN MED (13)	CLIN J PAIN (3)	EUR J PHYS REHAB MED(3)	TURK J PH MED REHAB (4)	BMC MUSCULOSKEL DIS(4)
3	SCAND J MED SCI SPOR(6)	AM FAM PHYSICIAN (2)	J BODY MOV THER (3)	ARCH RHEUMATOL (3)	PLOS ONE (3)
4	ARCH PHYS MED REHAB (5)	CURR RHEUMATOL REP (2)	REUMATISMO (3)	J PHYS THER SCI(3)	BMC MUSCULOSKEL DIS (2)
5	RHEUMATOL INT (5)	J AGING PHYS ACTIV (2)	TRIALS (3)	ARCH PHYS MED REHAB (2)	MED SCI SPORT EXER (2)

n: Number

INT J ENV RES PUB HE (*n* = 18), RHEUMATOL INT (*n* = 17), ARCH PHYS MED REHAB (*n* = 15), J CLIN MED (*n* = 14) and DISABIL REHABIL (*n* = 13) were the top five journals in terms of article count. Spain led contributions in INT J ENV RES PUB HE (13 publications), ARCH PHYS MED REHAB (5 publications), J CLIN MED (13 publications), and DISABIL REHABIL (*n* = 4). Türkiye and Spain had the highest contributions in RHEUMATOL INT (5 publications). Table 4 shows the leading five countries in these journals.

**Table 4:** Top five countries in the five most active journals

	Top five journals				
Rank	INT J ENV RES PUB HE	RHEUMATOL INT	ARCH PHYS MED REHAB	J CLIN MED	DISABIL REHABIL
1	Spain (13)	Spain (5)	Spain (5)	Spain (13)	Spain (4)
2	Portugal (2)	Türkiye (5)	Brazil (2)	United States (1)	Belgium (2)
3	Chile (1)	Brazil (2)	China (2)	-	Canada (2)
4	Netherlands (1)	Canada (2)	Türkiye (2)	-	Brazil (1)
5	Sweden (1)	Greece (2)	Australia (1)	-	Netherlands (1)

Data expressed as number

**Fig. 5:**
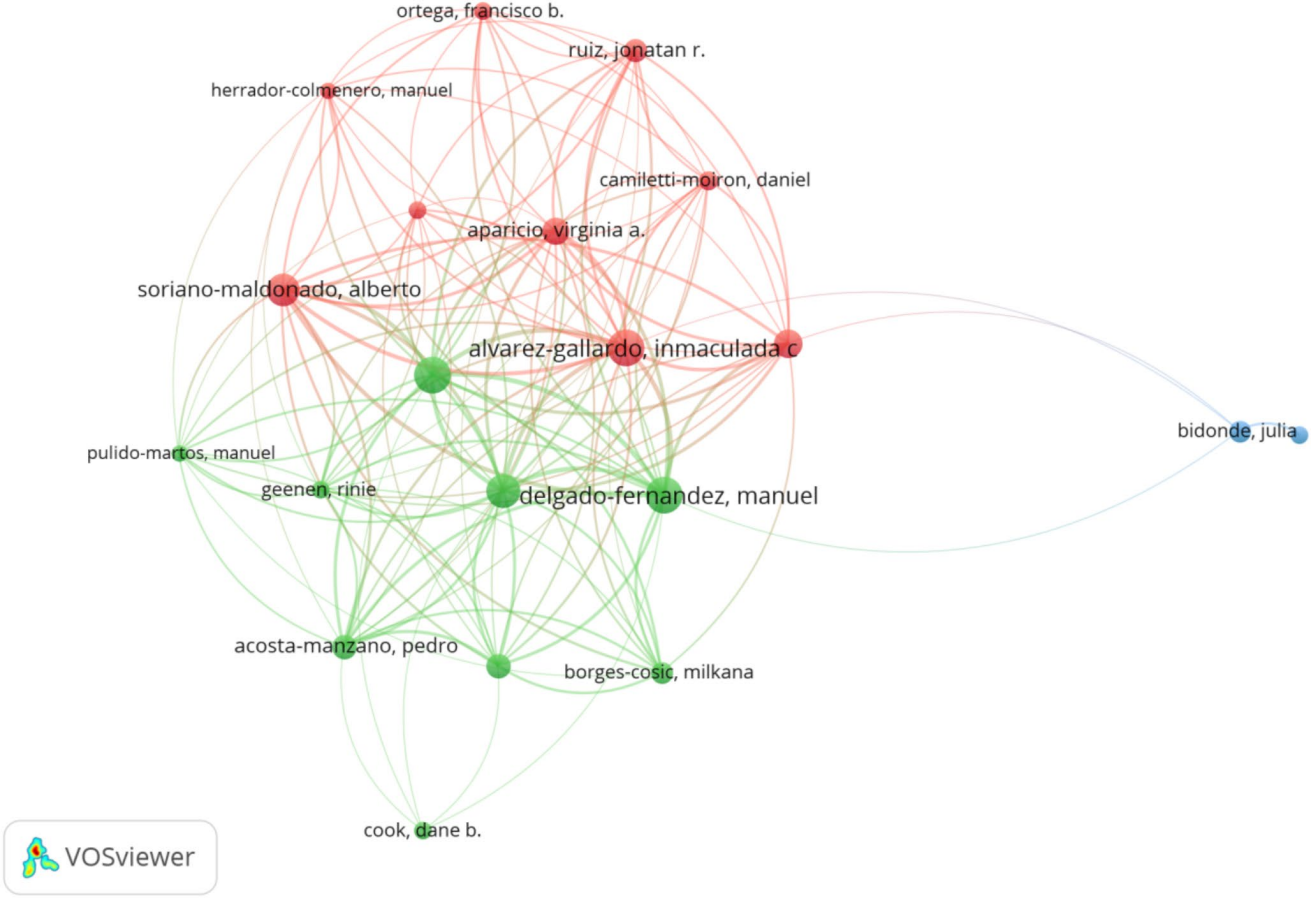
Author co-authorship network visualization map. Three clusters were created. Cluster 1 (9 authors) is presented in red; Cluster 2 (9 authors) is presented in green; and Cluster 3 (2 authors) is presented in blue

The institutions with the highest number of articles were Universidad de Extremadura (*n* = 25), University of Granada (*n* = 24), and Universidade do Estado de Santa Catarina (*n* = 15) (Table 5). Among the funding organizations, the Spanish Government (and = 53), the National Institutes of Health (*n* = 34), and the University of Granada (*n* = 24) were at the forefront (Table 6). Prolific authors in this field were Delgado-Fernández, M (*n* = 28), Segura-Jiménez, V (*n* = 27), and Alvarez-Gallardo, IC (*n* = 26) (Table 7). The author co-authorship network visualization map is shown in Fig. 5. Before visualization, an author’s minimum number of articles and citations were set to 5 and 50, respectively, resulting in 66 authors. Visualization was carried out according to the number of articles produced.

**Table 5:** Top five institutions in terms of publication count

Institution	Count
Universidad de Extremadura	25
University of Granada	24
Universidade do Estado de Santa Catarina	15
Universidad Rey Juan Carlos	12
University of Gothenburg	10

**Table 6:** Top five funding organizations by number of articles

Funding Organization	Count
Spanish Government	53
National Institutes of Health (NIH)	34
University of Granada	24
Swedish Rheumatism Association	16
Sahlgrenska University	13

**Table 7:** Top five prolific authors in fibromyalgia-exercise field

Author	Count	Percent
Delgado-Fernández, M	28	5.6%
Segura-Jiménez, V	27	5.4%
Alvarez-Gallardo, IC	26	5.2%
Estévez-López, F	23	4.6%
Soriano-Maldonado, A	22	4.4%

**Fig. 6:**
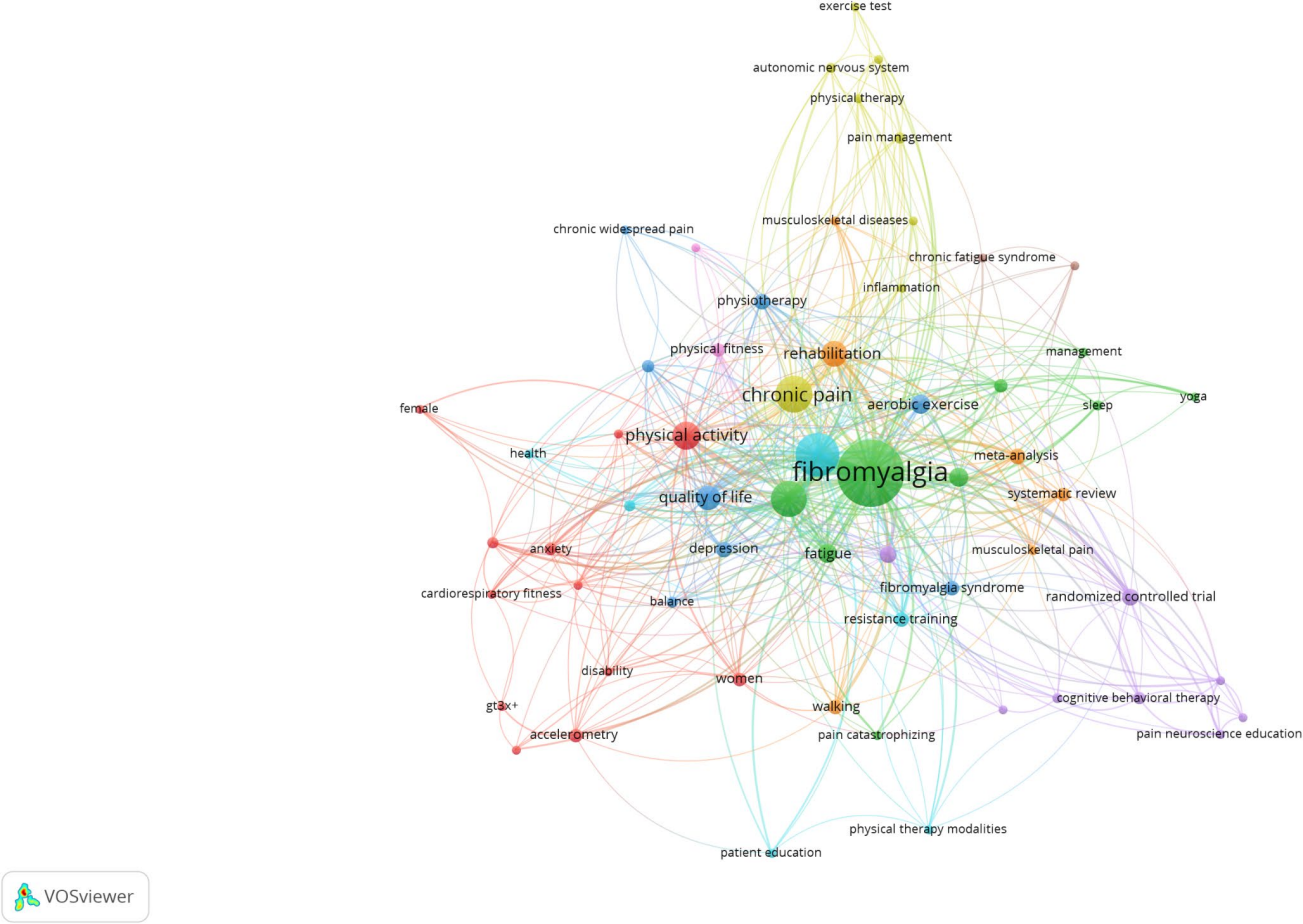
Author keywords co-occurrence network visualization map. Nine clusters were created. Cluster 1 (12 keywords) is presented in red; Cluster 2 (9 keywords) is presented in green; Cluster 3 (8 keywords) is presented in blue; Cluster 4 (8 keywords) is presented in light green; Cluster 5 (8 keywords) is presented in purple; Cluster 6 (6 keywords) is presented in light blue; Cluster 7 (6 keywords) is presented in orange; Cluster 8 (2 keywords) is presented in brown; and Cluster 9 (2 keywords) is presented in pink

In VOSviewer settings, 5 was entered as the minimum number of occurrences of a keyword, and 61 keywords met this requirement. The full counting method was used, and the five most frequently used keywords were determined as fibromyalgia (occurrences = 279; 34.19%), exercise (occurrences = 117; 14.33%), chronic pain (occurrences = 82; 10.04%), pain (occurrences = 81; 9.92%), and physical activity (occurrences = 49; 6.00%). The visualization was based on the total number of occurrences, and the visualization map is presented in Fig. 6.

## Discussion

This bibliographic study examined worldwide research trends concerning fibromyalgia and exercise from 2014 to 2023. A continuous rising trend in publications was noticed. High-income countries predominated in research contributions, with Spain at the forefront in publication volume and adjusted contributions relative to population and GDP. Reviews were cited in greater numbers than original articles, and publications indexed in SCIE and/or SSCI garnered much more citations than those in ESCI. The United Kingdom attained the highest average citations per publication.

The annual rise in publication counts highlights a growing global interest in the role of exercise in fibromyalgia management. This trend indicates an increased recognition of exercise as a non-pharmacological intervention, underscoring its significance in multidisciplinary care strategies [23]. The rise may also indicate a better comprehension of exercise’s efficacy in alleviating its diverse symptoms. Moreover, it emphasizes the increasing acknowledgment of exercise as an attainable and economical therapeutic alternative in accordance with global efforts to advance comprehensive and sustainable healthcare approaches [24, 25].

High-income nations, notably Spain, the United States, and Sweden contributed substantially to the field. This corresponds with overarching trends indicating that research productivity is associated with economic resources, strong research infrastructure, and the availability of financing. Spain’s dominance in this field likely signifies its strategic investment in public health and rehabilitative research. The restricted contributions from low- and middle-income countries underscore persistent differences in research capabilities and resource accessibility [26, 27].

The citation patterns demonstrate the profound effect of research published in high-impact journals. Journals indexed in SCIE and/or SSCI included a higher proportion of Q1 and Q2 categories, with 43.81% (*n* = 191) in Q1 and 38.3% (*n* = 167) in Q2. This distribution highlights the concentration of significant research in elite journals, indicating the elevated publication standards that prevail in this field. Articles indexed in SCIE and/or SSCI received much more citations than those in ESCI, highlighting the value of journal quality and indexing in enhancing research visibility and impact. This outcome signifies that SCIE journals had higher standards than ESCI ones and a larger number of articles [19].

Spain contributed 25.75% of publications, indicating its prominence in research prioritization and public health endeavors. The United States (15.09%) mirrors a formidable research infrastructure and substantial institutional support. Brazil (13.88%) and Türkiye (7.24%) underscore the increasing contributions of upper-middle-income countries. Despite its smaller population, Sweden (5.23%) demonstrates a robust dedication to significant research. The UK’s preeminence in average citations per article (107.08) indicates an emphasis on high-impact research. Countries like the UK and Norway show higher citation averages despite producing comparatively fewer papers, which may be attributed to several variables. These countries prioritize high-impact research via established financial structures, strategic collaborations, and institutional support emphasizing quality over quantity. Moreover, scholars from these nations often disseminate their findings in prestigious journals, thus enhancing the prominence and impact of their study. Additionally, international collaborations with elite universities globally may enhance the scope and influence of research from these countries.

Spain ranked first with 2.71 publications per million population, followed by Sweden at 2.46 and Norway at 1.81. These data points underscore the substantial production of smaller European countries concerning population size, demonstrating their efficacy in producing research relative to their demographic base [28]. This outcome indicates a prioritization of fibromyalgia in these areas, connecting national research initiatives with public health requirements. Spain again led with 5.71 publications per GDP unit, followed by Sweden (3.84) and Portugal (2.73). These findings highlight the efficient use of economic resources to produce high research output.

The five most active journals in the five most productive countries were detected as INT J ENV RES PUB HE and J CLIN MED in Spain, ARTHRIT CARE RES, and CLIN J PAIN in United States, ADV RHEUMATOL, EUR J PHYS REHAB MED, J BODY MOV THER, REUMATISMO, and TRIALS in Brazil, RHEUMATOL INT and TURK J PH MED REHAB in Türkiye, and ARTHRITIS RES THER and BMC MUSCULOSKEL DIS in Sweeden. According to these findings, certain journals seem to be gaining ground in particular nations. Journals may prioritize publications sourced from their home countries, which is consistent with previous investigations [26, 29].

INT J ENV RES PUB HE, RHEUMATOL INT, ARCH PHYS MED REHAB, J CLIN MED, and DISABIL REHABIL ranked as the top five journals based on article volume, accordingly. The results align with the subject’s integrity. Given the significance of exercise in managing fibromyalgia as a public health concern, both a public health journal and a general medicine journal emerged prominently. RHEUMATOL INT is an expected situation to be included in the list as a rheumatology journal. Two rehabilitation journals were prominent due to their association with exercise.

Our analysis indicates that the institutions with the highest publication counts were Universidad de Extremadura, University of Granada, and Universidade do Estado de Santa Catarina. The presence of Spanish institutions indicates an established national research emphasis in this domain, likely propelled by targeted funding and academic collaboration. The Spanish Government, the National Institutes of Health, and the University of Granada emerged as the principal funding organizations, signifying a substantial commitment to foster progress in this domain. These observations highlight the necessity of continuous institutional support and financing in promoting high-impact studies and international collaboration.

The implications of these results surpass academic contributions and underscore the necessity for strategic policy and funding initiatives. Considering the research concentration within a limited number of institutions, authorities should consider diversifying funding options to assist nascent research centers and under-represented areas. Forming international collaborations, especially with institutions from low- and middle-income countries, may augment shared knowledge and global influence. Furthermore, funding organizations should prioritize research in under-represented regions to close the gap between scientific discoveries and practical applications. Enhancing research infrastructure in under-represented locations and providing targeted incentives for early-career researchers may promote a more equitable and globally inclusive research environment.

This research has certain limitations. The literature review was conducted only via the WoS database. Limiting publications to original articles and reviews in English excluded publications in other languages or formats. This study focuses on publications from 2014 to 2023, so outcomes may vary over an extended timeframe. This analysis identified major contributors but did not examine comprehensive collaboration networks or co-authorship patterns, which could offer additional insights into global research dynamics.

This study extends previous bibliometric analyses in RHEUMATOL INT [19, 21, 26, 29–31] by thoroughly investigating global research trends with fibromyalgia and exercise over the last ten years. The current paper is distinguished by bibliometric analyses concentrating on exercise practices, which are prominent among non-pharmacological therapeutic alternatives in managing fibromyalgia. Given its low cost and efficacy, the number and quality of research on exercise procedures are anticipated to rise progressively. The current findings will assist researchers engaged in this domain.

## Conclusion

From 2014 to 2023, the quantity of papers on ‘fibromyalgia exercise’ exhibited a notable annual upward trend. High-income countries, particularly Spain, the United States, and Sweden made substantial contributions to the field, highlighting disparities in research capabilities. The fact that most publications came from high-income and upper-middle-income nations shows that health authorities in other countries should fund and support fibromyalgia exercise research. As a result of the multidisciplinary nature of the subject, public health, general medicine, rheumatology, and rehabilitation journals emerged as the most prolific sources of articles. This study identifies gaps in research outputs and under-represented regions, establishing a foundation for future investigations to enhance understanding and therapeutic strategies for fibromyalgia with respect to exercise. Cooperative initiatives, fair distribution of resources, and focused research on individual patient requirements are crucial for advancing global development in this field.

## Data Availability

Raw data can be provided upon request.

## Declarations

### Disclaimer

Authors state that no part of this manuscript, including the text and graphics, are copied or published elsewhere in whole or in part.
